# Inhibition of Lipid Accumulation in Skeletal Muscle and Liver Cells: A Protective Mechanism of Bilirubin Against Diabetes Mellitus Type 2

**DOI:** 10.3389/fphar.2020.636533

**Published:** 2021-01-25

**Authors:** Claudia A. Hana, Eva-Maria Klebermass, Theresa Balber, Markus Mitterhauser, Ruth Quint, Yvonne Hirtl, Antonia Klimpke, Sophie Somloi, Juliana Hutz, Elisabeth Sperr, Paulina Eder, Jana Jašprová, Petra Valášková, Libor Vítek, Elke Heiss, Karl-Heinz Wagner

**Affiliations:** ^1^ Department of Nutritional Sciences, Faculty of Life Sciences, University of Vienna, Vienna, Austria; ^2^ Department of Biomedical Imaging and Image-guided Therapy, Division of Nuclear Medicine, Medical University of Vienna, Vienna, Austria; ^3^ Ludwig Boltzmann Institute Applied Diagnostics, Vienna, Austria; ^4^ Institute of Medical Biochemistry and Laboratory Diagnostics, University General Hospital and 1^st^ Faculty of Medicine, Charles University, Prague, Czechia; ^5^ 4^th^ Department of Internal Medicine, University General Hospital and 1^st^ Faculty of Medicine, Charles University, Prague, Czechia; ^6^ Department of Pharmacognosy, University of Vienna, Vienna, Austria

**Keywords:** bilirubin, mild hyperbilirubinaemia, ectopic lipid accumulation, C2C12 skeletal muscle cells, HepG2 cells, [^18^F]FDG uptake, insulin resistance, lipid accumulation

## Abstract

Ectopic lipid accumulation in skeletal muscle and liver drives the pathogenesis of diabetes mellitus type 2 (DMT2). Mild hyperbilirubinaemia has been repeatedly suggested to play a role in the prevention of DMT2 and is known for its capacity to shape an improved lipid phenotype in humans and in animals. To date, the effect of bilirubin on lipid accumulation in tissues that are prone to ectopic lipid deposition is unclear. Therefore, we analyzed the effect of bilirubin on lipid accumulation in skeletal muscle and liver cell lines. C2C12 skeletal mouse muscle and HepG2 human liver cells were treated with physiological concentrations of free fatty acids (FFA) (0.5 mM and 1 mM) and unconjugated bilirubin (UCB) (17.1 and 55 µM). The intracellular presence of UCB upon exogenous UCB administration was confirmed by HPLC and the lipid accumulation was assessed by using Nile red. Exposure of both cell lines to UCB significantly reduced lipid accumulation by up to 23% (*p* ≤ 0.001) in HepG2 and by up to 17% (*p* ≤ 0.01) in C2C12 cells at 0.5 and 5 h under hypoglycaemic conditions. Simultaneously, UCB slightly increased FFA uptake in HepG2 cells after 0.5 and 5 h and in C2C12 cells after 12 h as confirmed by gas chromatographic analyses of the remaining FFA content in the incubation media. The effects of UCB on lipid accumulation and uptake were abolished in the presence of higher glucose concentrations. Monitoring the uptake of a radiolabeled glucose analogue [18F]FDG: (2-deoxy-2-[^18^F]fluoro-D-glucose) into both cell types further indicated higher glucose consumption in the presence of UCB. In conclusion, our findings show that UCB considerably decreases lipid accumulation in skeletal muscle and liver cells within a short incubation time of max. 5 h which suggests that mildly elevated bilirubin levels could lower ectopic lipid deposition, a major key element in the pathogenesis of DMT2.

## Introduction

Globally, the increase in diabetes mellitus type 2 (DMT2) is emerging with 463 million cases worldwide, a prevalence that tripled over the last 20 years and is projected to rise to 700 million cases in 2045 (International Diabetes Federation, 2019). Obesity and an increased fat mass are major contributors to the development of insulin resistance and DMT2. Many studies have shown that the distribution of body fat and particularly the deposition of lipids within non-adipose tissues drive insulin resistance. Indeed, ectopic lipid accumulation in tissues such as skeletal muscle and liver that are responsible for the majority of insulin stimulated glucose disposal drive the pathogenesis and progression of DMT2 (Shulman 2000; Moore et al., 2003; Goodpaster and Wolf 2004).

Prospective and retrospective human studies repeatedly found a protective role of mildly elevated blood bilirubin levels in DMT2 and a Mendelian Randomization study observed a causal risk reduction for DMT2 by mild hyperbilirubinaemia (Vítek 2012; Abbasi et al., 2015; Nano et al., 2016; Yang et al., 2019). Further, subjects with Gilbert´s syndrome (GS), an existing human model with constant mildly increased unconjugated blood bilirubin (UCB) levels have repeatedly been linked to a reduced fat mass, BMI and blood lipids (Wallner et al., 2013) and also exhibited a health beneficial glucose metabolic phenotype including a reduction in fasting glucose, insulin, C-peptide and insulin resistance (Mölzer et al., 2016; Khoei et al., 2018). This might suggest that mild hyperbilirubinaemia reduces the risk of DMT2 by protecting from disadvantageous, diabetes related changes in lipid metabolism. To date, data about bilirubin and its relation to ectopic lipid accumulation is scarce and limited to the liver, showing a decreased hepatic lipid content in mildly hyperbilirubinaemic mice (Hinds et al., 2017). However, experimental studies on lipid accumulation in skeletal muscle cells and hepatocytes, tissues that are prone to ectopic lipid deposition and simultaneously responsible for the majority of the insulin-stimulated glucose disposal are lacking. Therefore, this study aims for the first time to investigate the effect of UCB on intracellular lipid accumulation in C2C12 skeletal mouse muscle cells and HepG2 hepatoblastoma-derived cells.

## Materials and Methods

### Cell Culture and Treatment

C2C12, murine myoblast (ATCC Cat# CRL-1772, RRID:CVCL_0188) and HepG2, human hepatoblastoma cells (ATCC Cat# HB-8065, RRID:CVCL_0027) purchased from LGC Standards GmbH were routinely cultured in high glucose (25 mM) containing DMEM (D1145, Sigma-Aldrich, US) with 10% FBS (10270-098, Gibco, Thermo Fisher, US), 4 mM L-glutamine (G7513, Sigma-Aldrich, US) and 1% penicillin/streptomycin (P0781, Sigma-Aldrich, US) at 37°C, 5% CO_2_ under subconfluent conditions. Prior to experiments, HepG2 cells were grown to confluence for 2 days and confluent C2C12 cells were supplemented for another 6 days with 2% horse serum (10500-056, Gibco, Thermo Fisher, US) to allow differentiation into multinucleated myotubes. Cells were then treated with 0.5 mM or 1 mM BSA-coupled free fatty acids (FFA) (Svedberg et al., 1990) using palmitic acid (PA, P9767, Sigma-Aldrich, US), oleic acid (OA, O7501, Sigma-Aldrich, US) and linoleic acid (LA, L8134, Sigma-Aldrich, US) at a ratio of 2:1:2 which is consistent with the physiological FFA concentration and composition in human blood (Hodson et al., 2008). Simultaneously cells were exposed to 17.1 µM UCB (B412, Sigma-Aldrich, US), the threshold of the UCB blood concentration that characterizes GS (Wagner et al., 2018). In addition, 55 µM UCB was used for the principal lipid-based experiments to reflect the higher UCB concentrations in the liver compared to other tissues such as muscle (Zelenka et al., 2008) using an upper UCB concentration seen in sera of GS subjects. UCB was dissolved in DMSO (0.275% final DMSO concentration in all treatments) and then added to the BSA-containing media by shaking. Prior to the experiments, the solubility of UCB was routinely checked for possible UCB microprecipitate formation under the microscope. All experiments were performed in a darkened room. The control and reference for all treatments consisted of DMEM supplemented with 4 mM L-glutamine, 0.45 mM BSA and 0.275% DMSO. Cytotoxicity of the treatments was tested by MTT assay with 1 × 10^4^ C2C12 and 0.5 × 10^5^ HepG2 in 96 well plates. Formazan crystals were formed with 1 mg/ml MTT (M2128-1G, Sigma-Aldrich, US) in DMEM (37°C, 5% CO_2,_ 4 h), dissolved in 200 µL DMSO (37°C, 5% CO_2,_ 1 h) and measured at 485 nm using a plate reader (Fluorostar Optima, BMG Labtech, Germany).

### Cellular Unconjugated Bilirubin Uptake

The cellular UCB uptake and the remaining UCB concentration in the incubation media were analyzed by HPLC. Briefly, 4 × 10^5^ C2C12 and 4.5 × 10^6^ HepG2 cells were seeded into 100 × 20 mm cell culture dishes for treatment (10 ml). After exposure to UCB for 0.5 and 5 h, the supernatant was immediately frozen at −80°C. The cells were put on ice, washed with 10 ml PBS (4°C), collected using a cell scraper (5 ml PBS 4°C) and centrifuged at 179 g, 4°C for 5min. The cell pellet was stored in 1 ml PBS (4°C) at −80°C after adding N_2_.

The sample solutions for HPLC analysis consisted of 500 µL of UCB-non-treated media, 50 µL of UCB-treated media or 450 µL of cellular homogenate, disintegrated by ultrasound on ice as well as 50 µL of internal standard (5 mM mesobilirubin, Frontier Scientific, US) and the trace of BHT (antioxidant) (Sigma-Aldrich, US). UCB was extracted into 6 ml of methanol/chloroform/hexane (10/5/1 v/v/v) and concentrated into a small droplet of carbonate buffer (100 mM, pH 10) which was injected (50 µL) onto HPLC Agilent 1200 (CA, US) equipped with a diode-array detector. Bilirubin was then separated on the Luna C8 column (4.6 mm × 150 mm, 3 mM/100A, Phenomenex, CA, US). The mobile phase for the analysis contained methanol (450 g), water (300 g) and tetrabutylammonium hydroxide (7.5 ml) and its pH was adjusted by phosphoric acid (9–9.3). The signal was detected at 440 nm with 550 nm as the reference wavelength (Zelenka et al., 2008). The final amount of bilirubin in the medium was calculated as nmoL of UCB per mL of sample. The amount of protein in the cellular homogenate was measured by Bio-Rad Protein Assay (Bio-Rad, CA, US) using the microplate reader (Sunrise, Tecan, Austria). Concentration of bilirubin in cells was calculated as pmol of UCB per mg of protein.

### Lipid Accumulation and Uptake

Lipid accumulation was measured by the Nile red (NR) assay that selectively stains intracellular lipids. Therefore, 1 × 10^4^ C2C12 and 0.5 × 10^5^ HepG2 cells were seeded into a black 96 well µclear plate (Greiner, Austria) for treatment (final volume 200 µL). Then cells were fixed with 3% paraformaldehyde (F1635, Sigma-Aldrich, US) (24°C, 20min), stained with NR (3.3 µg/ml in PBS, 37°C, 5% CO2, 2 h) and rinsed with 100 µL PBS. NR fluorescence was measured from the bottom of the plate at ex: 485 nm and em: 590 nm, the background fluorescence was subtracted prior staining and NR fluorescence was adjusted by Bradford protein content (B6916, Sigma-Aldrich, US). Images of intracellular lipids were obtained with the confocal microscope Leica TCS SPE including the inverse microscope DMi8 and analyzed using the LAS X 2.0.014332 software from Leica.

Lipid accumulation at a single cell level was measured using flow cytometry after NR staining. C2C12 (0.2 × 10^6^) and HepG2 (0.6 × 10^6^) cells were seeded into a six well plate (Greiner, Austria) with 2 ml treatment solution. Cells were harvested with accutase (A6964, Sigma-Aldrich, US), collected in a 5 ml Polystyrene Round-Bottom Tube (352058, Corning, US), centrifuged (179 g, 5 min), washed with 1 ml PBS (37°C) and incubated in the dark for 15 min with 1 ml 0.75 µg/ml NR in PBS. Cells were centrifuged and analyzed in 400 µL NR solution. 50.000 cells were measured in FL2 excluding cell debris using a four-channel FACS CaliburTM flow cytometer (BD, Europe).

The cellular lipid uptake was analyzed indirectly by measuring the remaining FFA concentrations from the incubation media by gas chromatography. The lipid extraction was performed according to modified protocols from Folch and Metcalfe (Folch et al., 1957; Metcalfe et al., 1966). First, the sample solution consisting of 2 ml incubation media and 100 µL of internal standard (2 mM stearic acid in isopropanol) was extracted twice using 10ml/5 ml chloroform/methanol (2:1) for 20 min on ice while vortexing twice for 0.5 min. The lipophilic phases were collected (2,000 g for 10 min), pooled and evaporated with N_2_ (37°C). Then, saponification and methylation were performed using 1 ml methanolic NaOH (0.5 M NaOH and 0.27 mM BHT) and 1 ml BF3 (14% in methanol) respectively at 100°C for 5 min. The samples were immediately cooled on ice for 10 min and fatty acid methyl esters (FAMES) were collected twice in 2 and 1.5 ml hexane by shaking for 20 min. The hexane phases were pooled, evaporated with N_2_ (37°C) and dissolved in 200 ml hexane. Samples were analyzed with an auto system gas chromatograph (Perkin Elmer Clarus 500, US), a Rtx-2330 30 m × 0.25 mm i.d. silica column, a flame ionization detector (270°C) and helium as a carrier gas. FAMES, 1 µL per sample were injected at an initial injector temperature of 250°C. The initial oven temperature was set to 90°C and was increased 3 times. First to 150°C by 13.0°C/min, then to 180°C by 2.9°C/min with an isothermal period of 5 min and finally to 230°C by 4.0°C/min and an isothermal period of 7 min. FFAs were quantified based on a six point calibration curve (200µg/ml-800 µg/ml) using a five component FAMES Mix Standard ME28 (8691.1, Carl Roth, Germany). The concentration of the FFAs was adjusted according to recovery of the internal standard.

### Glucose Uptake Using [^18^F]FDG

We analyzed the cellular uptake of 2-deoxy-2-[^18^F]fluoro-D-glucose ([^18^F]FDG), a radiolabeled glucose analogue, which allows to mimic glucose uptake. [^18^F]FDG is GLUT-dependently transported into cells mainly via GLUT1 (Wiebe 2001) which is predominantly expressed in C2C12 (Calderhead et al., 1990) and HepG2 cells (Takanaga et al., 2008) and allows to monitor basal glucose uptake. [^18^F]FDG uptake was investigated at a single time point and by real-time kinetic measurements over 60 min using LigandTracer^®^ Yellow (Ridgeview Instruments, Sweden) (Balber et al., 2019). Briefly, C2C12 (0.16 × 10^6^) and HepG2 (1.2 × 10^6^) cells were seeded into a six well plate and incubated with 4 ml UCB treatment solution for 5 and 24 h followed by 2 h glucose starving in the presence of UCB. 150kBq of [^18^F]FDG were added to the cells for 1 h at 37°C, subsequently, supernatant and cells (accutase) were collected and measured with a gamma counter (Wizard^2^ ™ 3”, PerkinElmer, USA) for 30 s. Results were expressed relative to a reference adding [^18^F]FDG to PBS instead of treatment and cells. For the real-time kinetic [^18^F]FDG measurements, 0.8 × 10^6^ C2C12 and HepG2 cells were seeded onto one half of a 100 × 20 mm cell culture dish, the opposite side was used as a reference. Cells were treated analogue to the six well assay and cell associated radioactivity was measured starting directly after addition of [^18^F]FDG for 60 min.

### Statistical Analysis

Statistical analysis and calculations were completed using IBM SPSS 24 (IBM SPSS Statistics, RRID: SCR_019096). Data distribution was checked based on histograms and box plots. For parametric data One-Way ANOVA and for non-parametric data Kruskal-Wallis test were performed. Alpha was set at *p* ≤ 0.05 according to Bonferroni.

## Results

### Exogenous Administration of Unconjugated Bilirubin Increased the Intracellular Unconjugated Bilirubin Content and Was Not Cytotoxic

**FIGURE 1:**
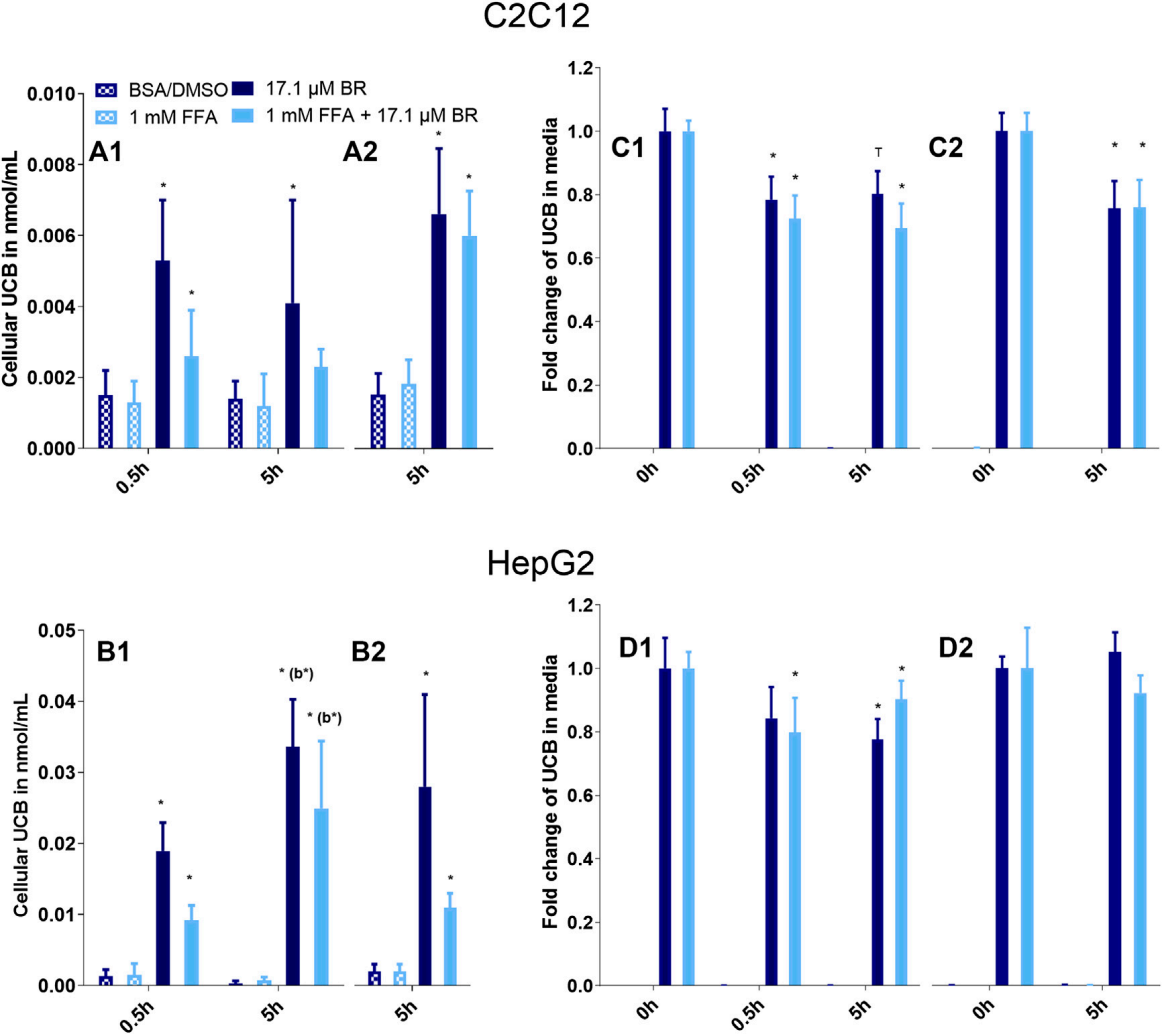
UCB significantly increased the cellular UCB content of **(A)** C2C12 and **(B)** HepG2 cells and UCB concentration in the incubation media of **(C)** C2C12 and **(D)** HepG2 cells dropped significantly after 0.5 and 5 h. Cells were incubated under (**1**) hypoglycaemic conditions or (**2**) with glucose. Data are mean ± SD of four independent replicates, *p ≤ 0.05 and T *p* ≤ 0.1 compared to cells treated without UCB, compared to media prior incubation or compared to *b* = the preceding incubation time.

**FIGURE 2:**
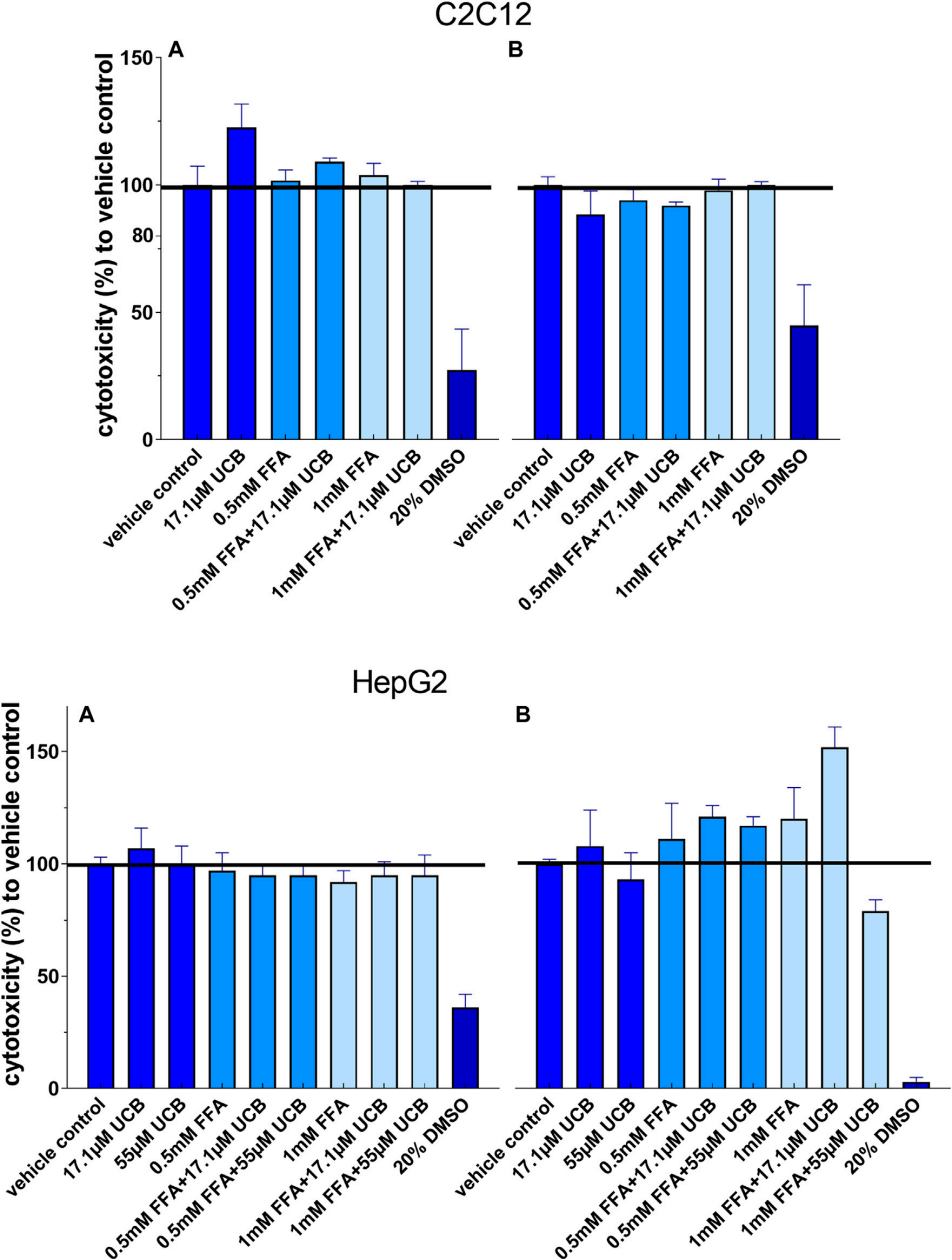
None of the treatments was cytotoxic to C2C12 and HepG2 cells: Cells were incubated **(A)** at hypoglycaemic conditions for 12 h (C2C12) and 5 h (HepG2) or **(B)** with glucose for 24 h. Absorbance 485 nm, data are mean ± SD of at least n = 2 (each in sextuplicate).

The exposure of C2C12 and HepG2 cells to 17.1 µM UCB significantly increased the intracellular UCB concentration after 0.5 and 5 h (Figures 1A, B). Consistently, the UCB concentration in the incubation media of both cell lines decreased significantly after 0.5 and 5 h (Figures 1C, D). None of the treatments was cytotoxic to C2C12 or HepG2 cells compared to the vehicle control (Figure 2).

### Unconjugated Bilirubin did Not Change Lipid Accumulation in C2C12 and HepG2 Cells in the Presence of Glucose

**FIGURE 3:**
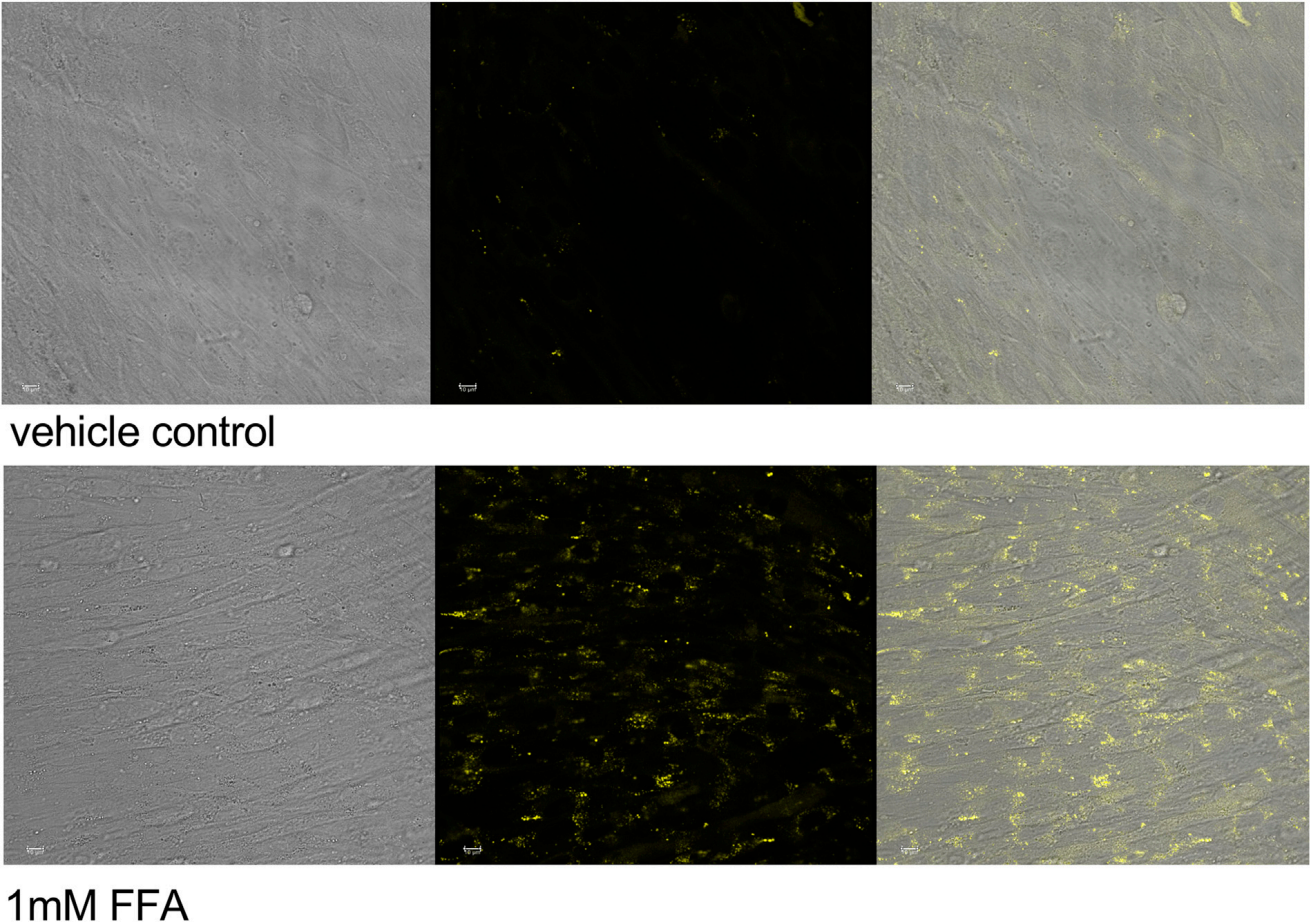
Lipid accumulation and lipid droplet formation in C2C12 myotubes Cells were incubated for 5 h, stained with NR and viewed by fluorescence confocal microscopy. Transmission **(left)**, fluorescence **(center) **images and overlay **(right)**, ex: 488 nm, em: 509–560 nm, no adjustment of gain.

**FIGURE 4:**
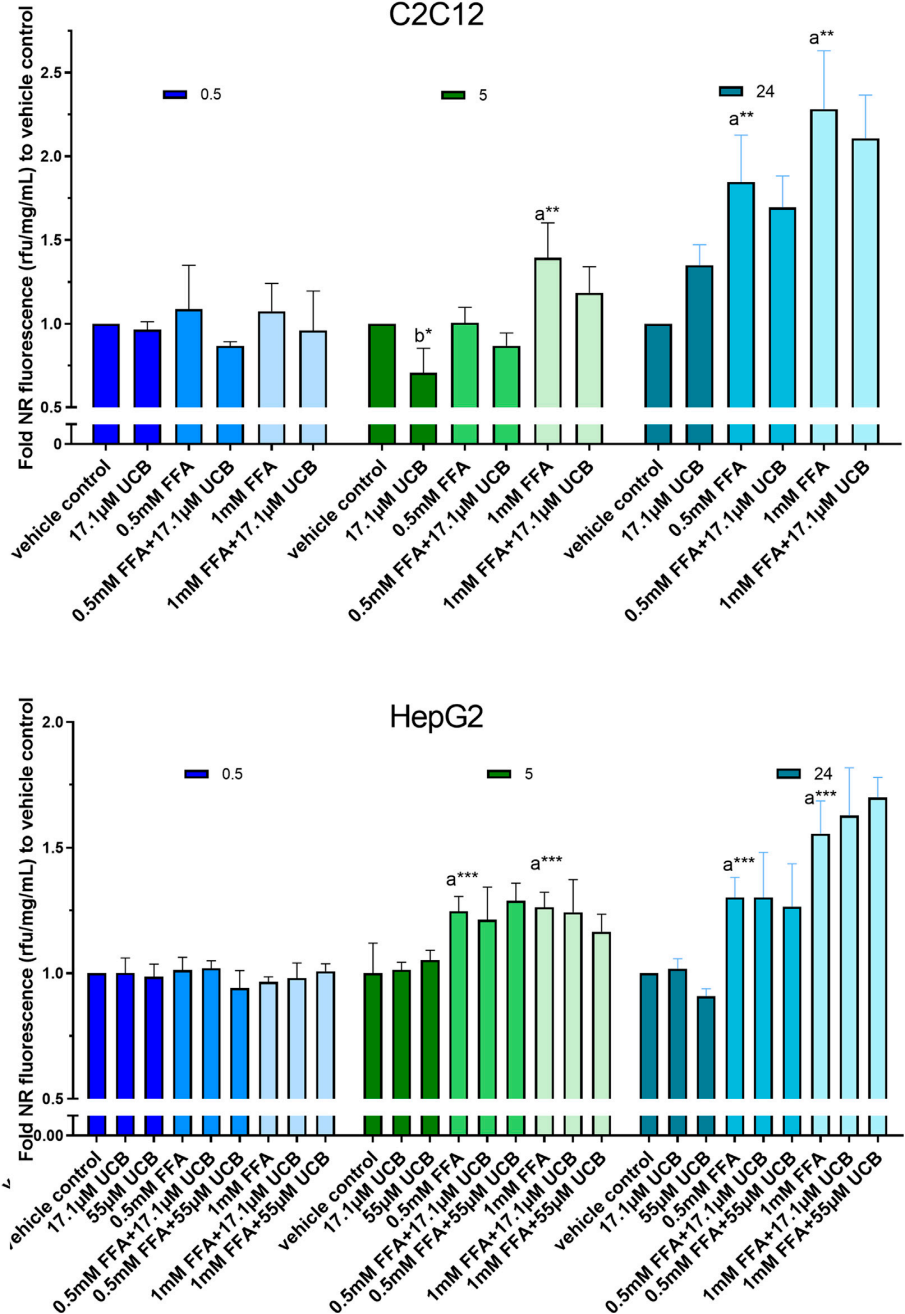
UCB had no significant effect on lipid accumulation in C2C12 and HepG2 cells in the presence of glucose. Cells were exposed to treatments for 0.5, 5 and 24 h. NR fluorescence was adjusted by protein content (rfu/mg/mL) and compared to vehicle control. Data are mean ± SD of n = 3 (each in triplicate). Significant differences according to Bonferroni (***p ≤ 0.001, **p ≤ 0.01, *p ≤ 0.05) of a = compared to vehicle control and b = UCB treated vs. untreated cells.

First, intracellular lipids and lipid droplet formation upon FFA incubation for 5 h were confirmed via confocal microscopy (Figure 3). In a second step, lipid accumulation was quantified via NR assay. C2C12 and HepG2 cells were exposed to 0.5mM and 1 mM FFAs which significantly increased the content of intracellular lipids in both cell lines compared to vehicle control after 5 and 24 h of incubation. However, the treatment with UCB did not significantly change the basal or the FFA-driven lipid accumulation in C2C12 and HepG2 cells. We only observed a reproducible but non-significant slight reduction in lipid accumulation in all FFA treatments in C2C12 cells (Figure 4). This was somewhat unexpected, however, the treatments were performed in presence of glucose. It is known that skeletal muscle and liver preferentially rely on glucose as energy source upon sufficient glucose availability but switch from glucose toward lipids at hypoglycaemic conditions. Further, people with GS beside having health beneficial lipid blood and anthropometric biomarkers (Wallner et al., 2013; Wagner et al., 2015), also exhibited beneficial changes in glucose metabolism (decreased fasting glucose, insulin and C-peptide) in a more recent study (Mölzer et al., 2016). This could suggest that UCB has effects on the glycaemic response of our cell model masking or overriding changes in lipid metabolism. Therefore, we assessed in a next step basal glucose uptake into C2C12 and HepG2 cells upon UCB treatment by using the radiolabeled glucose analogue [^18^F]FDG.

### Unconjugated Bilirubin Mildly Increased Cellular [^18^F]FDG Uptake in C2C12 and HepG2 Cells

**FIGURE 5:**
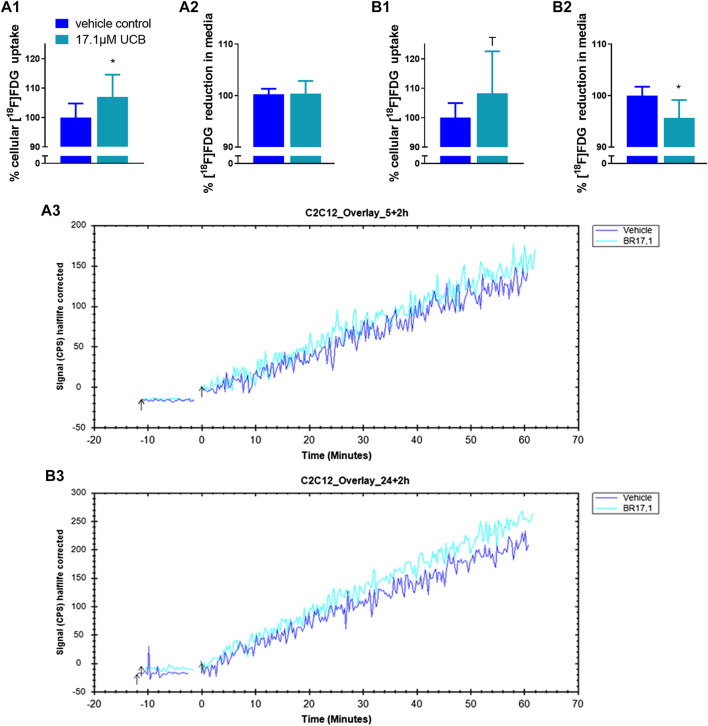
UCB changed [^18^F]FDG uptake in C2C12 cells. (**1**) Cellular uptake (%) of [^18^F]FDG and (**2**) reduction (%) of [^18^F]FDG in the supernatant determined by gamma counting after UCB incubation compared to vehicle control (100%) at **(A)** 5 h and **(B)** 24 h. Data are mean ± SD of n = 3 (each in triplicate). *p ≤ 0.05, T *p* ≤ 0.1. **(3)** Representative [^18^F]FDG kinetics (0–60min) after UCB/vehicle treatment.

**FIGURE 6:**
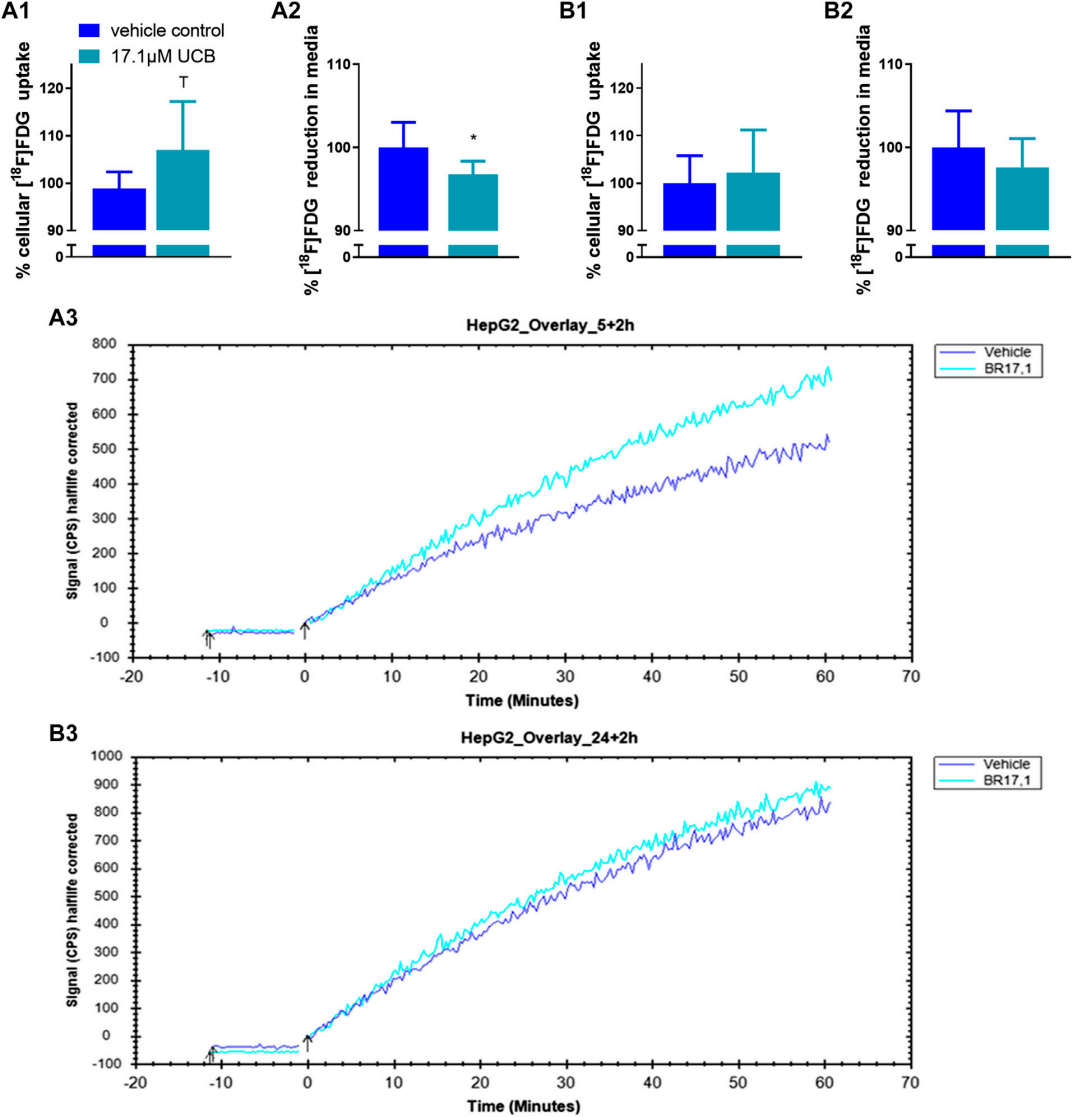
UCB changed [^18^F]FDG uptake in HepG2 cells. (**1**) Cellular uptake (%) of [^18^F]FDG and (**2**) reduction (%) of [^18^F]FDG in the supernatant determined by gamma counting after UCB incubation compared to vehicle control (100%) at **(A)** 5 h and **(B)** 24 h. Data are mean ± SD of n = 3 (each in triplicate). *p ≤ 0.05, T *p* ≤ 0.1. **(3)** Representative [^18^F]FDG kinetics (0–60 min) after UCB/vehicle treatment.

In C2C12 cells, UCB significantly increased the intracellular [^18^F]FDG content by 7% after 5 h and by 8% after 24 h (Figures 5A1,B1). Simultaneously, UCB lowered the [^18^F]FDG concentration in the incubation media significantly by 4% after 24 h (Figure 5B2). Consistent with these observations, cell associated radioactivity derived from [^18^F]FDG decay was slightly higher in real-time kinetic measurements after exposure of C2C12 cells to UCB for 5 and 24 h (Figure 5A3,B3). In HepG2 cells, UCB increased the intracellular [^18^F]FDG content by trend by 8% and significantly reduced the [^18^F]FDG concentration in the media by 7% only after 5 h of incubation (Figure 6A1,A2). In real-time kinetic measurements, cell associated radioactivity derived from [^18^F]FDG decay was again higher after exposure to UCB for 5 and 24 h (Figure 6A3,B3).

### Unconjugated Bilirubin Decreases Lipid Accumulation in C2C12 and HepG2 Cells at Hypoglycaemic Conditions

In a second batch of experiments the effect of UCB on lipid accumulation was investigated in the absence of glucose with incubation times up to 5 h. We decided to remove glucose 1) due to the effects of UCB on [^18^F]FDG uptake in our cell model and 2) to challenge lipid catabolism since glucose deprived conditions stimulate the oxidative metabolism as shown in HepG2 cells (Weber et al., 2002). Prior to the lipid accumulation experiment, we confirmed that the UCB uptake was largely unaltered in the absence of glucose compared to glucose conditions and that the treatments were not cytotoxic to the cells (Figures 1A1,B1,C1,D1,2A).

**FIGURE 7:**
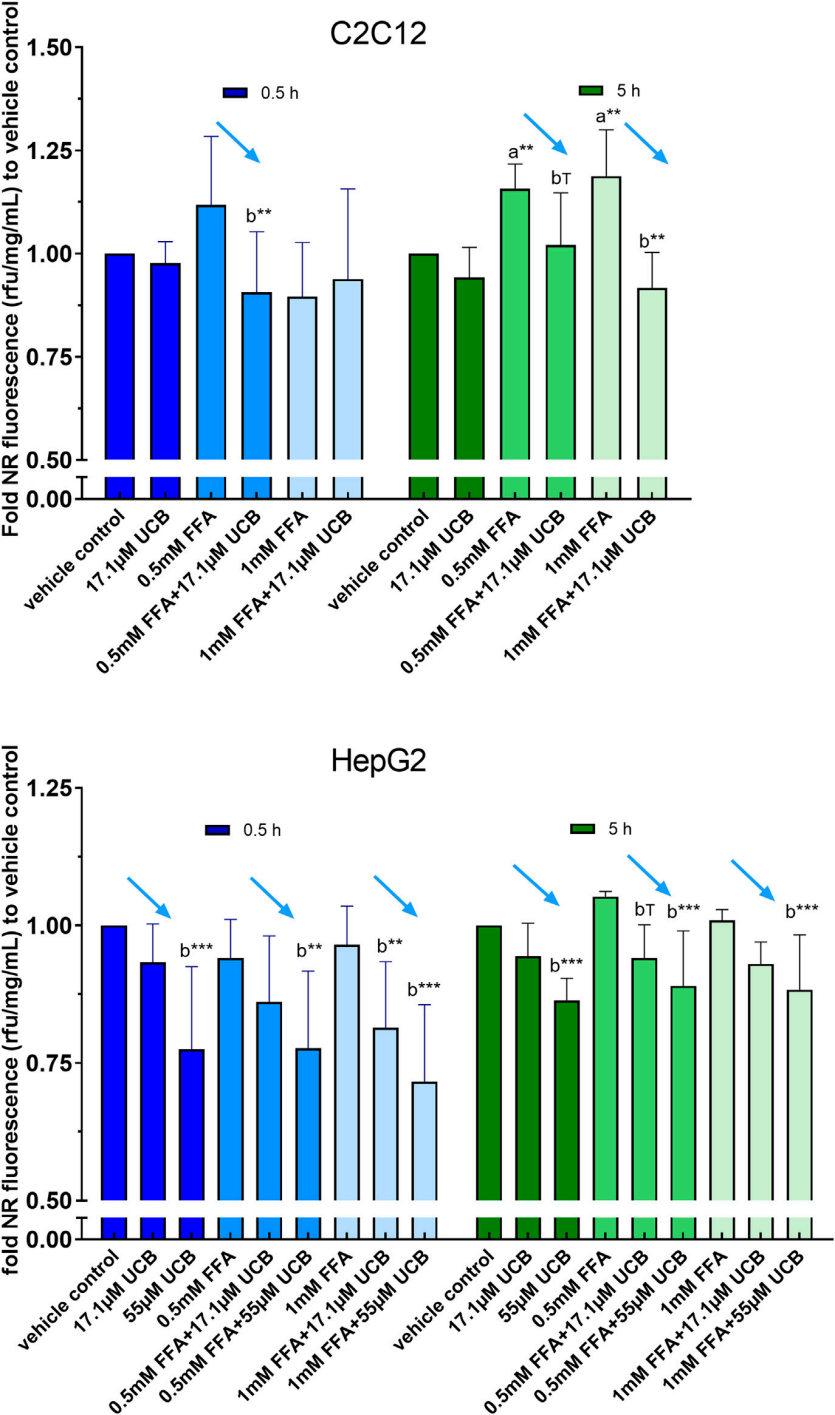
UCB significantly decreased lipid accumulation in C2C12 and HepG2 cells at hypoglycaemic conditions. Cells were exposed to treatments for 0.5 and 5 h. NR fluorescence was adjusted by protein content (rfu/mg/mL) and compared to vehicle control. Data are mean ± SD of n = 3 (each in quadruplicate). ***p ≤ 0.001, **p ≤ 0.01, T *p* ≤ 0.1 of *a* = compared to vehicle control and *b* = compared to UCB treated vs. untreated cells according to Bonferroni.

**FIGURE 8:**
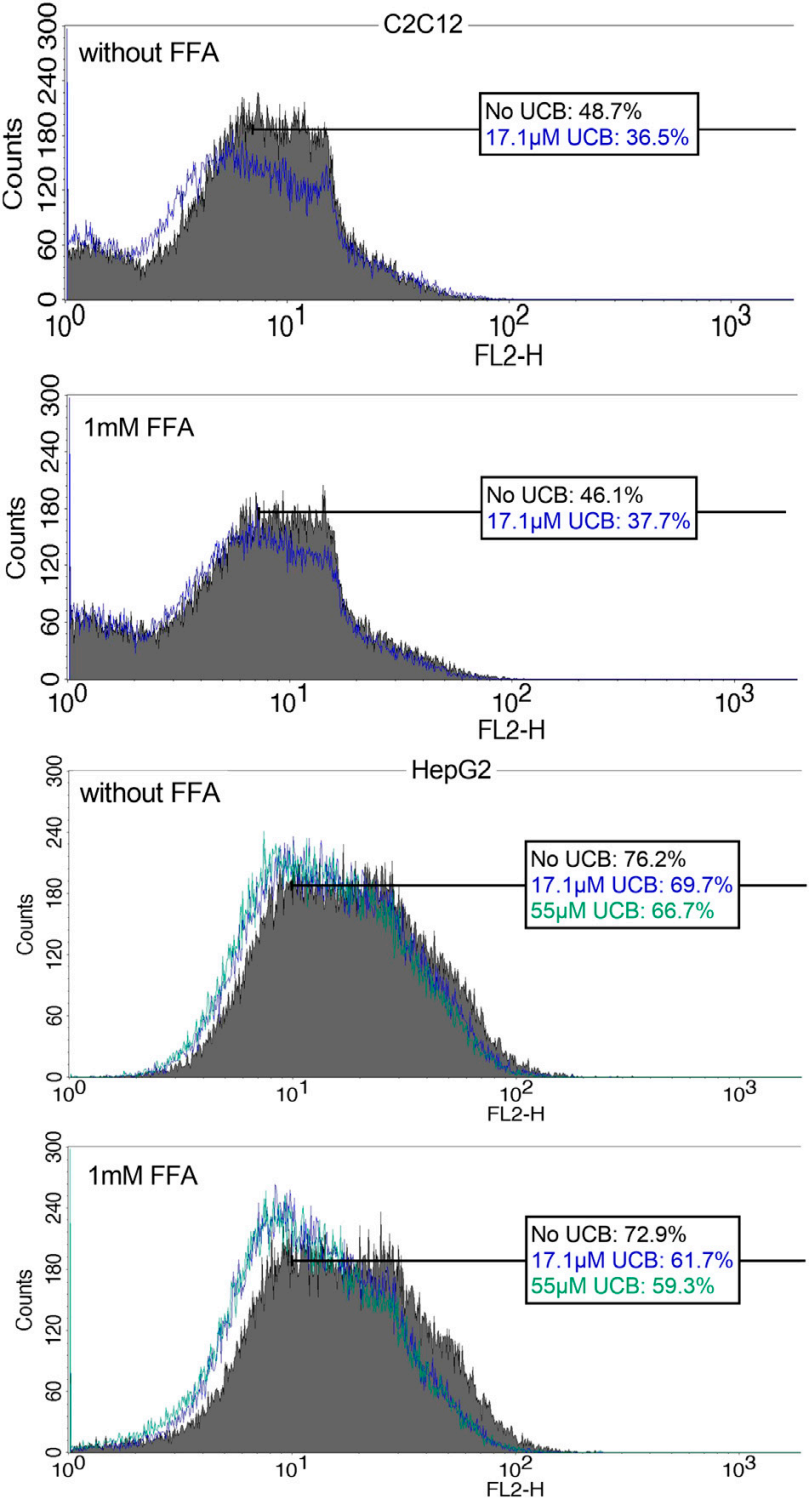
NR fluorescence indicates a decreased lipid accumulation in C2C12 and HepG2 cells upon UCB exposure. Cells were exposed to the respective treatments for 5 h at hypoglycaemic conditions. Cell associated fluorescence was analyzed by flow cytometry (FL2). The blue (17.1 µM UCB) and green (55 µM UCB) histograms show a shift to the left toward decreased NR fluorescence intensity in comparison to the gray histogram (without UCB). The boxes show the % of cells with a higher NR fluorescence *intensity* after incubation without UCB, with 17.1 µM or 55 µM UCB. Representative histogram of Supplementary Tables S2 of 50,000 cells.

C2C12 cells exposed to 17.1 µM UCB significantly decreased the intracellular lipid accumulation by up to 17% (*p* ≤ 0.01) compared to vehicle controls at 0.5 and 5 h. In HepG2 cells, UCB significantly decreased lipid accumulation in all treatments after 0.5 and 5 h by up to 23% (*p* ≤ 0.001), whereas 55 µM UCB further decreased the lipid accumulation compared to 17.1 µM UCB, indicating a dose dependency (Figure 7). Lipid accumulation was also decreased after UCB treatment in C2C12 and HepG2 cells using flow cytometry at hypoglycaemic conditions, in contrast to treatments with glucose (Figures 7, 8, Supplementary Tables S1, S2).

### Unconjugated Bilirubin Slightly Increased Cellular Lipid Uptake

**FIGURE 9:**
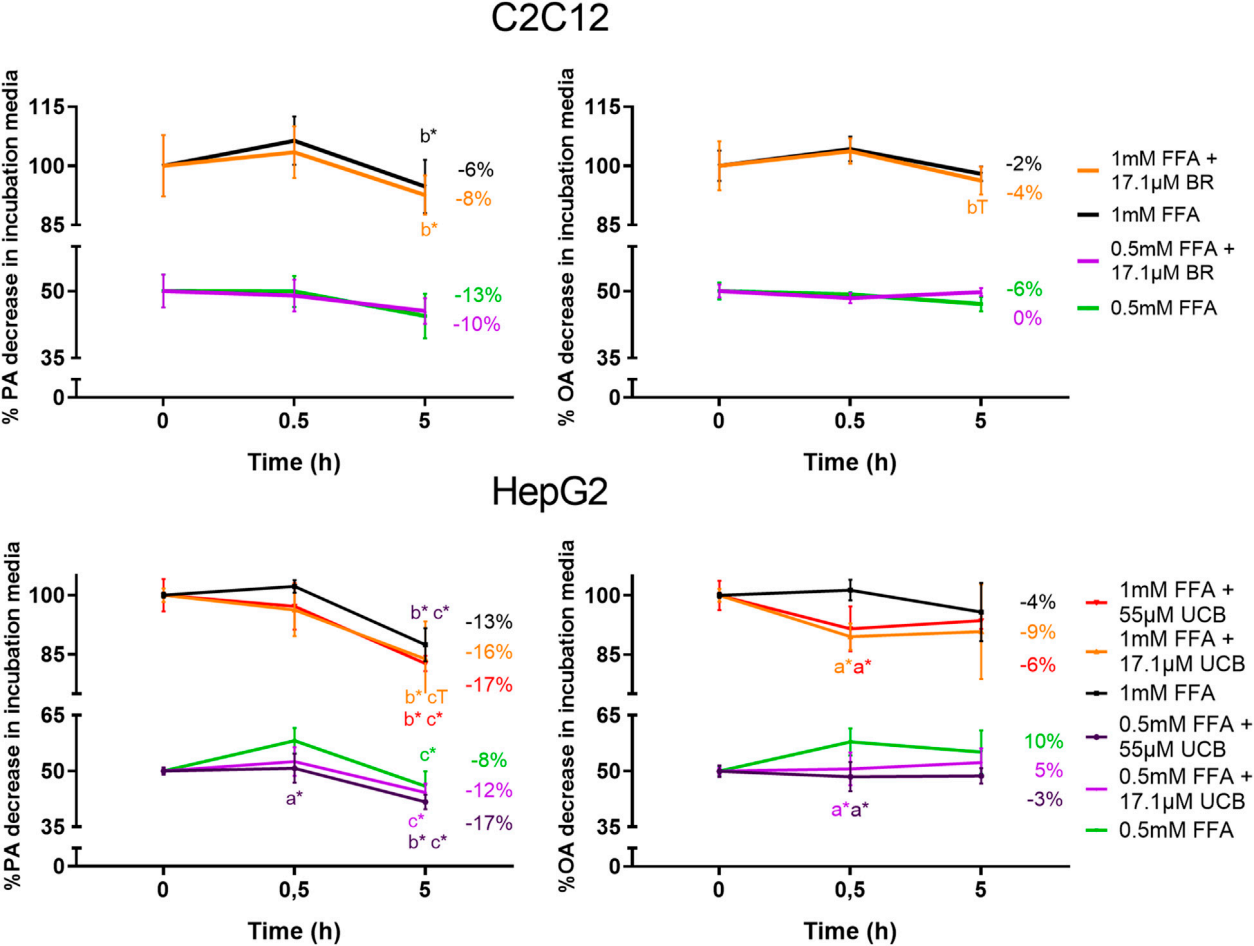
UCB slightly increased the FFA uptake of HepG2 cells and did not significantly change the FFA uptake of C2C12 cells. Cells were exposed to the respective treatments for 0.5 and 5 h at hypoglycaemic conditions. The FFA uptake was assessed indirectly by measuring the PA and OA concentrations in the supernatant of both cell lines by gas chromatography. Data are mean ± SD of n = 3 (each in triplicate). *p ≤ 0.05, T *p* ≤ 0.1 of *a* = compared to UCB treated vs. untreated cells, of *b* = compared to the preceding incubation time and of *c* = compared to the initial concentration of the incubation media.

To exclude a decreased lipid uptake as a reason for the decreased lipid accumulation upon UCB treatment, we analyzed the remaining FFA concentrations in the incubation media by gas chromatography. UCB did not change the concentration of PA and OA in the treatments of C2C12 cells, which indicates that UCB did not change the cellular FFA uptake after 0.5 and 5 h under hypoglycaemic conditions (Figure 9). Further investigations of FFA uptake up to 12 h showed that UCB slightly reduced the FFA concentrations by approximately 5% in the incubation media of C2C12 cells at hypoglycaemic conditions in contrast to treatments with glucose (Supplementary Figures S1, S2). In detail, PA dropped by 22% and OA by 10% significantly in the presence of UCB and 1 mM FFA, whereas the drop of PA by 17% and OA by 6% was not significant in the absence of UCB (Supplementary Figure S1). In HepG2 cells, PA and OA concentrations were consistently lower (x̅≈8%) in the presence of UCB over all treatments and incubation times which was significant at 0.5 h. Thus, this data not only excludes that UCB decreases lipid uptake, it also indicates that UCB slightly increases FFA uptake in both cell lines.

## Discussion

Over the last decade, accumulating evidence has been suggesting a role of mildly elevated systemic bilirubin concentrations in the prevention of metabolic diseases and particularly in DMT2. Epidemiologic studies repeatedly showed a protection against DMT2 at mildly increased blood bilirubin levels (Abbasi et al., 2015; Nano et al., 2016). In case control studies, GS individuals with constant mild hyperbilirubinaemia revealed a reduced fat mass, BMI and blood lipids next to improved insulin resistance, fasting glucose, insulin and C-peptide levels (Wallner et al., 2013; Mölzer et al., 2016). Mice with diet induced obesity displayed an improved insulin sensitivity and reduced body weight and fat mass when treated with bilirubin (Dong et al., 2014; Liu et al., 2015; Gordon et al., 2020). Genetically modified mice with mild hyperbilirubinaemia are resistant to high fat diet induced hepatic steatosis, obesity, hypertriglyceridaemia, glucose intolerance, hyperglycaemia and hyperinsulinaemia due to an increased PPARα activity (Hinds et al., 2017). This evidence indicates that mildly elevated bilirubin levels have a strong DMT2-preventive potential via shaping an improved lipid phenotype. However, the mechanisms of this lipid based diabetes-protective capacity of UCB remain unclear.

The pathogenesis of DMT2 involves ectopic lipid accumulation in tissues that account for most of the insulin-dependent glucose disposal. Studies about the effects of UCB on ectopic lipids are rare, only focused on the liver and show a reduced diet induced hepatic lipid content in the presence of mild hyperbilirubinaemia in mice (Hinds et al., 2017). However, to date there are no experimental studies about the effect of UCB on ectopic lipid deposition in skeletal muscle cells and hepatocytes, cell types that are responsible for the majority of the whole-body glucose uptake. Thus, this study focused on the UCB driven changes of lipid accumulation in C2C12 skeletal muscle cells and HepG2 hepatocytes.

Mild hyperbilirubinaemic conditions (17.1 µM UCB) significantly decreased the intracellular lipid accumulation by up to 17% (*p* ≤ 0.01) in C2C12 skeletal mouse muscle cells after 0.5 and 5 h. In HepG2 cells, exposure to mildly elevated UCB concentrations in the medium (17.1 and 55 µM) significantly reduced the lipid accumulation by up to 23% (*p* ≤ 0.001) dose dependently and consistently in all FFA treatments at 0.5 and 5 h of incubation (Figure 7). Simultaneously, a slightly decreased PA and OA concentration in the UCB containing incubation media indicated that UCB slightly increased FFA uptake in both cell lines (Figure 9, Supplementary Figure S1). This confirms that the lipid content in the presence of UCB is reduced not based on a decrease in lipid uptake but rather due to a reduction of intracellular lipids. This strong UCB-induced reduction in lipid accumulation by up to 23% within a short incubation period of max. 5 h suggests that mild hyperbilirubinaemia might considerably lower ectopic lipid accumulation.

In contrast to these experiments that were performed with hypoglycaemic media, UCB had no effect on lipid accumulation or uptake in both cell lines when using glucose at a concentration recommended for both cell lines (Figure 4, Supplementary Figure S2, Table S1). Under hypoglycaemic fasting conditions PPARα and AMPK are activated to upregulate the cellular lipid oxidative metabolism and lipid uptake (Kersten et al., 1999; Towler and Hardie 2007). Thus while in the presence of glucose, our cell model might preferentially metabolize glucose for energy production, the hypoglycaemia activates PPARα and AMPK and forces the cells into the oxidative lipid metabolism. We and other groups showed that the beneficial metabolic profile in mild hyperbilirubinaemia might be PPARα and AMPK dependent (Mölzer et al., 2016; Gordon et al., 2020) and UCB was shown to be an activator of PPARα (Stec et al., 2016). This might explain the differences of lipid accumulation and lipid uptake at varying glucose concentrations (Figures 4, 7, 9, Supplementary Figures S1, S2) and suggests an increased lipid oxidation as a reason for the UCB-induced reduction of lipid accumulation in muscle and liver cells.

Mild hyperbilirubinaemia has been associated with beneficial changes in the blood glucose profile in humans and in mice (Liu et al., 2015, Mölzer et al., 2016, Hinds et al., 2017). Hence, it is possible that UCB increased the glycaemic response of our cell model in the presence of glucose masking or overriding changes in lipid metabolism. UCB had indeed effects on the [^18^F]FDG uptake in both cell lines which was one of the main reasons why we continued our experiments under hypoglycaemic conditions. These experiments showed a slightly increased [^18^F]FDG uptake in C2C12 and HepG2 cells, indicating a rise of mainly GLUT1-dependent glucose uptake (Figures 5, 6). To our knowledge there is no other data to date about the effects of UCB on glucose uptake. However, there is one study that observed a significantly increased GLUT4 expression in the muscle cell membrane of bilirubin-treated mice (Dong et al., 2014).

This is the first study that reports a UCB driven decrease in lipid accumulation in skeletal muscle and liver cells, types of tissues that are most affected by ectopic lipid deposition. Lipid accumulation was investigated at varying physiological FFA concentrations considering the level of glucose and was simultaneously compared to the lipid uptake. Another strength of this study was that the uptake and the intracellular presence of UCB was confirmed by HPLC (Figure 1). For future experiments it is necessary to explore the effect of UCB on ectopic lipid deposition in animals or humans to further support our results.

## Conclusion

Our findings show that UCB considerably decreases lipid accumulation in skeletal muscle and liver cells by up to 23% within a short incubation time of max. 5 h. This suggests that mild hyperbilirubinaemia could lower ectopic lipid deposition, a major key element in the pathogenesis of DMT2. Thus, the reduced ectopic lipid accumulation in skeletal muscle and liver cells could indeed, at least partly explain the reduced insulin resistance in GS and the protective role of mild hyperbilirubinaemia in DMT2.

## Data Availability Statement

The raw data supporting the conclusion of this article will be made available by the authors, without undue reservation.

## Author Contributions

CAH designed the project and the experiments, performed experiments and statistical analysis and wrote the manuscript. K-HW. conceived the original idea, designed and supervised the project and revised the manuscript. E-MK and TB designed and performed glucose uptake experiments and revised manuscript. MM contributed his radiopharmaceutical expertize, supervised radioactive uptake experiments and revised the manuscript. RQ, YH, AK, JH, SS, PE, ES, JJ, and PV performed experiments. LV contributed the UCB uptake method and revised the manuscript. EH provided support with cell culture expertize and revised the manuscript. All authors provided final approval of the manuscript.

## Funding

This project was funded by the Austrian Hochschuljubiläumsstiftung der Stadt Wien, the FWF Stand-Alone Project P 29608 and RVO VFN64165/2020 from the Czech Ministry of Health.

## Conflict of Interest

The authors declare that the research was conducted in the absence of any commercial or financial relationships that could be construed as a potential conflict of interest.

## Abbreviations

DMT2, diabetes mellitus type 2; [^18^F]FDG, 2-deoxy-2-[^18^F]fluoro-D-glucose; FFA, free fatty acids; GS, Gilbert’s syndrome; LA, linoleic acid; NR, Nile red; OA, oleic acid; PA, palmitic acid; UCB, unconjugated bilirubin.
