# Supplementary material for: Inhibition of Lipid Accumulation in Skeletal Muscle and Liver Cells: A Protective Mechanism of Bilirubin Against Diabetes Mellitus Type 2
Source: Front Pharmacol. 2021 Jan 25;11:636533. doi: 10.3389/fphar.2020.636533 (PMC7868327; doi:10.3389/fphar.2020.636533)
Supplement: Supplementary file 1 [file datasheet1.pdf]

Supplementary Material

C2C12

Lipid accumulation by flow cytometry									
**Treatments**	**0.5h**			**5h**			**24h**		
**BSA**	1.00 ±	0.03		1.00 ±	0.02		1.00 ±	0.05	
**17.1µM UCB**	0.97 ±	0.08	n.s.	1.03 ±	0.05	n.s.	1.01 ±	0.08	n.s.
**0.5mM FFA**	1.00 ±	0.06	n.s.	1.15 ±	0.07	0.005	1.19 ±	0.09	0.000
**0.5mM FFA + 17.1µM UCB**	0.94 ±	0.07	n.s.	1.20 ±	0.08	n.s.	1.19 ±	0.07	n.s.
**1mM FFA**	1.02 ±	0.08	n.s.	1.27 ±	0.08	0.000	1.39 ±	0.07	0.000
**1mM FFA + 17.1µM UCB**	1.00 ±	0.09	n.s.	1.22 ±	0.12	n.s.	1.35 ±	0.09	n.s.

HepG2

Lipid accumulation by flow cytometry									
**Treatments**	**0.5h**			**5h**			**24h**		
**BSA**	1.00 ±	0.10		1.00 ±	0.08		1.00 ±	0.10	
**17.1µM UCB**	0.99 ±	0.09	n.s.	0.94 ±	0.15	n.s.	0.96 ±	0.11	n.s.
**55µM UCB**	0.95 ±	0.09	n.s.	0.99 ±	0.14	n.s.	0.92 ±	0.08	n.s.
**0.5mM FFA**	0.90 ±	0.06	n.s.	1.03 ±	0.14	n.s.	1.17 ±	0.18	n.s.
**0.5mM FFA + 17.1µM UCB**	0.84 ±	0.12	n.s.	0.93 ±	0.06	n.s.	1.05 ±	0.16	n.s.
**0.5mM FFA + 55µM UCB**	0.99 ±	0.31	n.s.	0.88 ±	0.05	n.s.	1.08 ±	0.24	n.s.
**1mM FFA**	0.94 ±	0.11	n.s.	1.14 ±	0.07	n.s.	1.44 ±	0.16	0.000
**1mM FFA + 17.1µM UCB**	0.93 ±	0.16	n.s.	0.94 ±	0.18	n.s.	1.43 ±	0.21	n.s.
**1mM FFA + 55µM UCB**	0.88 ±	0.15	n.s.	0.95 ±	0.18	n.s.	1.39 ±	0.23	n.s.

Table S1: Lipid accumulation in C2C12 and HepG2 single cells in the presence of glucose. Cells were analysed using flow cytometry. The geometric mean fluorescence (FL2) of 50,000 NR-stained cells per treatment was compared to vehicle control. Data are mean ±SD of n=3 (each in duplicate). n.s.=non significant.

C2C12

Lipid accumulation by flow cytometry		
**Treatments**	**0.5h**	**5h**
**BSA**	1.00 ± 0.18	1.00 ± 0.03
**17.1µM UCB**	1.04 ± 0.13 n.s.	0.88 ± 0.06 n.s.
**0.5mM FFA**	0.93 ± 0.14 n.s.	0.87 ± 0.07 n.s.
**0.5mM FFA + 17.1µM UCB**	0.87 ± 0.16 n.s.	0.76 ± 0.09 n.s.
**1mM FFA**	0.97 ± 0.11 n.s.	0.87 ± 0.07 n.s.
**1mM FFA + 17.1µM UCB**	0.85 ± 0.13 n.s.	**0.74 ± 0.05 0.045**

HepG2

Lipid accumulation by flow cytometry		
**Treatments**	**0.5h**	**5h**
**BSA**	1.00 ± 0.06	1.00 ± 0.05
**17.1µM UCB**	0.91 ± 0.08 n.s.	0.98 ± 0.09 n.s.
**55µM UCB**	0.90 ± 0.08 n.s.	0.92 ± 0.07 n.s.
**0.5mM FFA**	0.97 ± 0.13 n.s.	1.09 ± 0.06 n.s.
**0.5mM FFA + 17.1µM UCB**	0.81 ± 0.13 n.s.	0.91 ± 0.14 n.s.
**0.5mM FFA + 55µM UCB**	0.80 ± 0.09 n.s.	1.06 ± 0.10 n.s.
**1mM FFA**	0.92 ± 0.11 n.s.	1.05 ± 0.10 n.s.
**1mM FFA + 17.1µM UCB**	0.85 ± 0.11 n.s.	**0.84 ± 0.08 0.096**
**1mM FFA + 55µM UCB**	0.77 ± 0.08 n.s.	0.93 ± 0.15 n.s.

Table S2: Lipid accumulation in C2C12 and HepG2 single cells. Both cell lines were exposed to hypoglycaemic conditions and analysed using flow cytometry. The geometric mean fluorescence (FL2) of 50,000 NR-stained cells per treatment was compared to vehicle control. Data are mean ±SD of n=3 (each in duplicate). n.s.=non significant.

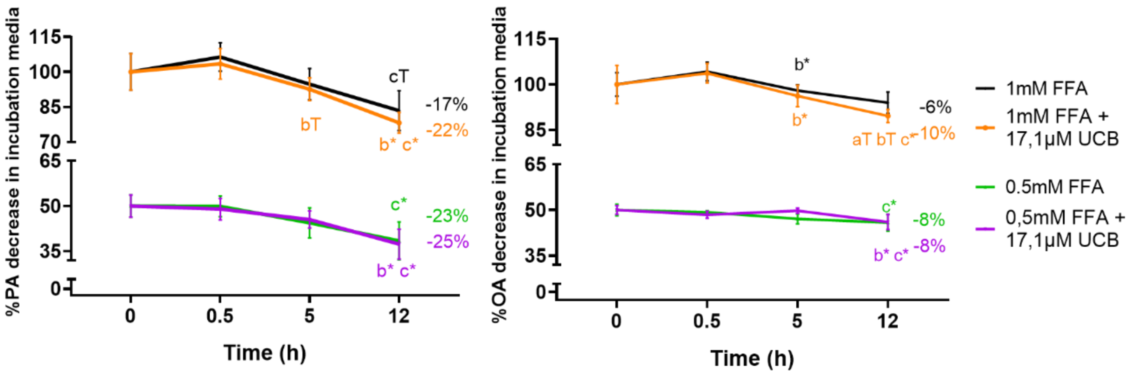

Figure S1: UCB slightly increased FFA uptake of C2C12 cells after 12h of incubation at hypoglycaemic conditions. FFA uptake was assessed indirectly by measuring the PA and OA concentrations in the supernatant of both cell lines by gas chromatography. Data are mean ±SD of n=3 (each in duplicate). *p≤0.05 and T p≤0.1 of a = compared UCB treated vs. untreated cells, of b = compared to the preceding incubation time and of c = compared the initial concentration of the incubation media.

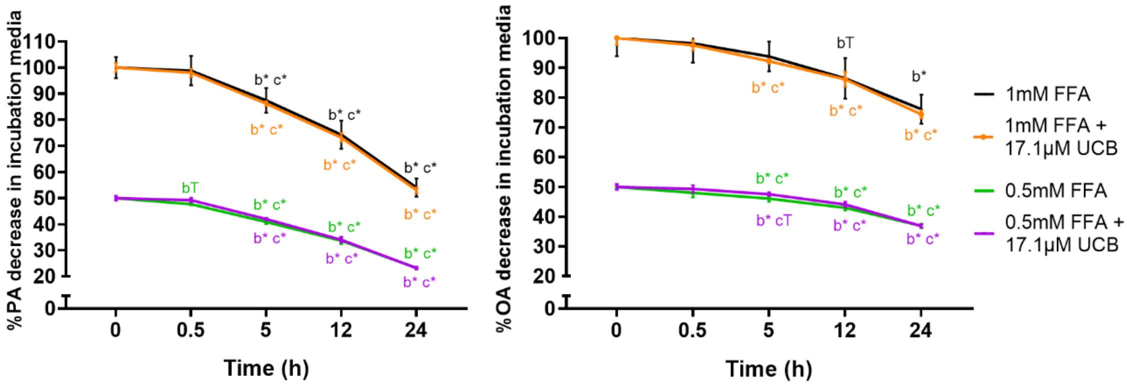

Figure S2: UCB did not change the FFA uptake of C2C12 during 24h of incubation when glucose was present. FFA uptake was assessed indirectly by measuring the PA and OA concentrations in the supernatant of both cell lines by gas chromatography. Data are mean ±SD of n=3 (each in duplicate). *p≤0.05 and T p≤0.1 of a = compared to UCB treated vs. untreated cells, of b = compared to the preceding incubation time and of c = compared to the initial concentration of the incubation media.
